# CONN-NLM: A Novel CONNectome-Based Non-local Means Filter for PET-MRI Denoising

**DOI:** 10.3389/fnins.2022.824431

**Published:** 2022-05-30

**Authors:** Zhuopin Sun, Steven Meikle, Fernando Calamante

**Affiliations:** ^1^School of Biomedical Engineering, The University of Sydney, Sydney, NSW, Australia; ^2^Faculty of Medicine and Health, The University of Sydney, Sydney, NSW, Australia; ^3^Brain and Mind Centre, The University of Sydney, Sydney, NSW, Australia; ^4^Sydney Imaging, The University of Sydney, Sydney, NSW, Australia

**Keywords:** diffusion MRI, PET-MRI, connectome, tractography, connectivity, image processing, denoising

## Abstract

**Background:**

Advancements in hybrid positron emission tomography-magnetic resonance (PET-MR) systems allow for combining the advantages of each modality. Integrating information from MRI and PET can be valuable for diagnosing and treating neurological disorders. However, combining diffusion MRI (dMRI) and PET data, which provide highly complementary information, has rarely been exploited in image post-processing. dMRI has the ability to investigate the white matter pathways of the brain through fibre tractography, which enables comprehensive mapping of the brain connection networks (the “connectome”). Novel methods are required to combine information present in the connectome and PET to increase the full potential of PET-MRI.

**Methods:**

We developed a CONNectome-based Non-Local Means (CONN-NLM) filter to exploit synergies between dMRI-derived structural connectivity and PET intensity information to denoise PET images. PET-MR data are parcelled into a number of regions based on a brain atlas, and the inter-regional structural connectivity is calculated based on dMRI fibre-tracking. The CONN-NLM filter is then implemented as a post-reconstruction filter by combining the nonlocal means filter and a connectivity-based cortical smoothing. The effect of this approach is to weight voxels with similar PET intensity and highly connected voxels higher when computing the weighted-average to perform more informative denoising. The proposed method was first evaluated using a novel computer phantom framework to simulate realistic hybrid PET-MR images with different lesion scenarios. CONN-NLM was further assessed with clinical dMRI and tau PET examples.

**Results:**

The results showed that CONN-NLM has the capacity to improve the overall PET image quality by reducing noise while preserving lesion contrasts, and it outperformed a range of filters that did not use dMRI information. The simulations demonstrate that CONN-NLM can handle various lesion contrasts consistently, as well as lesions with different levels of inter-connectivity.

**Conclusion:**

CONN-NLM has unique advantages of providing more informative and accurate PET smoothing by adding complementary structural connectivity information from dMRI, representing a new avenue to exploit synergies between MRI and PET.

## Introduction

Magnetic resonance imaging (MRI) and positron emission tomography (PET) provide complementary information that can be exploited to enhance *in-vivo* imaging examinations of the neurological system. MRI can provide high spatial resolution ranging from mm to sub-mm clinically (Shah, 2019). In neuroimaging applications, MRI offers remarkable versatility with multiple sequence types to examine structural, functional, and physiological characteristics of the brain. Alternatively, PET can be used to visualise and quantify biochemically-specific metabolic processes with very high sensitivity (Cherry et al., 2003). Clinical hybrid PET/MRI systems have been developed to combine the advantages of each modality through simultaneous acquisition of MRI and PET data (Chen et al., 2018). Integrated PET/MRI enables parallel acquisition of structural, perfusion, metabolic, and functional data, which is useful in research and may lead to useful clinical applications in neurological and psychiatric disorders.

In order to realise the significant potential of hybrid PET/MRI, complementary information can be used to improve the quality of the data from each modality, for example to perform artefact correction, enhance image quality, and improve diagnostic accuracy. MRI has been used in various ways to inform PET data processing, such as with MRI-based PET-MR motion correction (Chun et al., 2012) and incorporating MR priors into the PET reconstruction (Somayajula et al., 2005; Mehranian et al., 2017). In the post image reconstruction phase, MR-guided image filtering can help correct the partial volume effect and optimise the PET denoising process (Grecchi et al., 2017). PET images with improved quality may lead to more accurate qualitative and quantitative assessments.

The vast majority of methods developed to improve PET image quality using MRI information have focused on conventional structural MRI, i.e., T1- and T2-weighted imaging. For instance, in combination with anatomical information in T1-weighted images, a non-local smoothing method has been shown to improve the PET image quality and quantitative accuracy (Gao et al., 2018). Besides T1 and T2, diffusion MRI (dMRI) can provide a wealth of information about microstructure and connectivity, but its use in PET-MRI studies has been very limited. In particular, dMRI can investigate the white matter pathways of the brain through fibre tractography, allowing for comprehensive mapping of the brain structural connectivity networks (Jeurissen et al., 2019). Importantly, white matter connectivity can be associated with abnormalities detected with PET. For example, for imaging neurodegenerative disorders, advances in novel PET tracers targeting beta-amyloid and tau allow visualisation and quantification of neuronal dysfunction (Bischof et al., 2019). By examining both PET and dMRI in-vivo, tau-related white matter alterations were also reported in studies based on diffusion tensor imaging (DTI) (Jacobs et al., 2018; Wen et al., 2021). Among the affected white matter pathways in Alzheimer’s disease, DTI findings showed that long association fibres connecting distant brain regions were mostly impacted (Mito et al., 2018). Similarly, studies on human post-mortem tissue reported associations between white matter degeneration and pathological changes in Alzheimer’s disease (McAleese et al., 2017). Despite the presence of highly associated information from MRI and PET, very few studies have proposed new methods to exploit these synergies, likely due to the complexities involved in incorporating the connectivity information contained in fibre-tracking into PET post-processing (Calamante, 2017).

Our study presents the development of a method for denoising PET data based on dMRI-derived structural connectivity information. The method is called CONNectome-based Non-Local Means (CONN-NLM) filter^1^ (Sun et al., 2021). The proposed CONN-NLM method integrates connectivity information into the nonlocal means (NLM) weighting of PET voxel neighbourhoods (Buades et al., 2005). We evaluated the proposed method using realistic PET-MRI simulations and illustrated its use in clinical examples of co-registered tau PET/dMRI data from the Alzheimer’s Disease Neuroimaging Initiative Phase 3 (ADNI3).^2^

## Theory

### CONNectome-Based Non-local Means

#### Non-local Means Filter

The non-local means (NLM) filter was proposed to perform nonlocal averaging of all pixels in an image (Buades et al., 2005). The method can be used to reduce PET noise by calculating the weighted average of voxel values, where the weight is derived from an intensity similarity measure within a relatively large (non-local) search window. The NLM filter smooths voxel intensity value *x*_*i*_ by performing a weighted average of all voxel values *x*_*j*_ in a search window *N(i)* according to the similarity of patches around *x*_*i*_ and *x*_*j*_ (Buades et al., 2005):


(1) $$N\,L\,\left(x_{i}\right)=\sum_{j\in N\,\left(i\right)}w_{i\,j}\times x_{j}$$

(2) $$w_{i\,j}=\frac{1}{Z\,\left(i\right)}\times e\,x\,p\,\left(-\frac{{||\vec{x_{\text{i}}}-\vec{x_{\text{j}}}||}_{2,a}^{2}}{h^{2}}\right)$$

(3) $$Z\,\left(i\right)=\sum_{j}e\,x\,p\,\left(-\frac{{||\vec{x_{\text{i}}}-\vec{x_{\text{j}}}||}_{2,a}^{2}}{h^{2}}\right)$$

where *w*_*ij*_ is the intensity similarity weight, $\vec{x_{\text{i}}}$denotes a vector of intensity values within the square patch centred at voxel *i*, and $\vec{x_{\text{j}}}$denotes a vector of intensity values within the square patch centred at voxel *j*, and ||…|| _2,_*_*a*_* represents the L_2_ norm convolved with a Gaussian kernel of standard deviation *a*, which is used as similarity distance (Buades et al., 2005). The similarity between $\vec{x_{\text{i}}}$ and $\vec{x_{\text{j}}}$ is determined by a decreasing function of the Gaussian-weighted Euclidean distance, such that the Euclidean distance of the centre voxels is given more weight than the surrounding voxels. The variation in weight with respect to Euclidean distance is determined by *h*, a tuning parameter that eventually determines the smoothing strength: the larger the h, the more smoothing that is applied. The size of patches, *M*×*M*, and the similarity window, *N*×*N*, can be user defined. Typical neighbouring patches are 3 × 3 pixels in a similarity window of 11 × 11 pixels (Chan et al., 2014). *Z(i)* in Eq. (3) performs normalisation to ensure the sum of weights, ∑_*j*_*w*_*ij*_ = 1.

#### Distant/Local Connectivity

**FIGURE 1:**
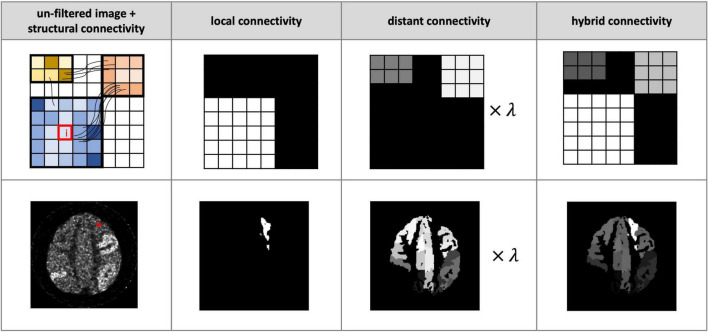
Illustration of proposed hybrid connectivity weighting. Each column from left to right: the noisy images without postfiltering plus structural connectivity information (schematically indicated by the connecting streamlines), the local connectivity, the structural connectivity-derived distant connectivity, and the hybrid connectivity weighting. *Top row*: a schematic demonstration of the computation of distant connectivity and local connectivity for smoothing voxel *i* (indicated by a red box). These two matrices are combined to form a hybrid connectivity weighing. *Bottom row*: a realistic example of smoothing voxel *i* (indicated by *) in a noisy positron emission tomography (PET) image. λ is set to 0.5 in both examples.

A common way to summarise the connectivity information is via the connectome, which in our case corresponds to a connectivity matrix representing the structural connectivity between pairs of regions in a brain parcellation. Such connectivity matrices are often used for subsequent graph theoretical analysis in connectomics (Fornito et al., 2013). In this study, we combined a tractography-based connectome (to represent the distal connectivity) with an intra-parcellation connectivity term (to represent the local connectivity), to create a hybrid local/nonlocal connectome model, similar to the model used in Hammond et al. (2013) for EEG smoothing (Figure 1). To compute the connectivity between a given voxel *i* in node *P_a_* of the parcellation and voxel *j* in node *P*_*b*_(i.e.,*i* ∈ *P*_*a*_,*j* ∈ *P*_*b*_), the proposed hybrid connectivity parameter *A*_*ij*_ is expressed as:


(4) $$A_{i\,j}=\{\begin{array}{c} A^{l\,o\,c\,a\,l}=1,P_{a}=P_{b}\\ A^{d\,i\,s\,t}=\lambda \times S\,C_{a\,b},P_{a}\neq P_{b} \end{array}$$

where *A^local^* is assumed to be uniform (with a normalised value of 1) for all voxels belonging to the same node, *A^dist^* is derived from the tractography-based structural connectivity (SC) matrix, and the parameter λ regulates the balance between distant and local connectivity. Streamlines generated from tractography are assigned to each node and the SC is generated using MRtrix3^3^ – see section “Diffusion Simulation and Fibre-Tracking Processing” for further tractography details. The SC matrix is log transformed and normalised to have a range between 0 and 1. For the results shown here, the 170 node AAL3 atlas (Rolls et al., 2020) was selected – note, however, that the choice of parcellation can be adapted according to the specific application. The resulting SC matrix measures the connectivity between each node pair: each element of the matrix (also referred to as an “edge”) corresponds to the SC between the corresponding pair of nodes (e.g., matrix element *SC*_*ab*_ corresponds to the SC between nodes *a* and *b* of the parcellation).

#### CONN-NLM Filter

**FIGURE 2:**
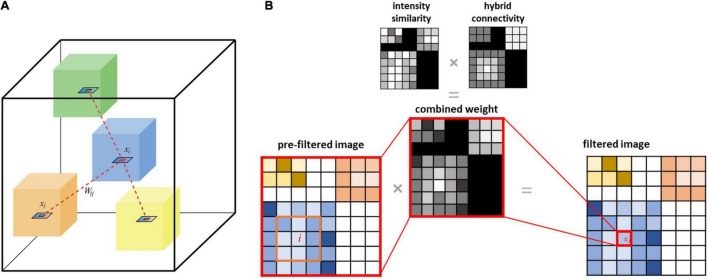
**(A)** Schematic illustration of the proposed connectome-based NLM filter applied to voxel intensity *x*_*i*_ (inside the blue cube), which incorporates connectivity (both local and distant) and intensity similarity information. Each coloured cube schematically represents a node of the brain parcellation; the dashed lines between the blue cube and other cubes represent the inter-nodal (distal) structural connectivity. **(B)** The filtered value for any given voxel *i* is computed as the weighted sum of values for every brain voxel. This weighting is based on voxel-wise PET intensity similarity and connectivity strengths between each pair of nodes in the parcellation (distant connectivity) and intra-node distance measurements (local connectivity).

Modified versions of the NLM filter can be implemented by incorporating structural information from CT and MRI to denoise PET (Chan et al., 2014; Gao et al., 2021). In these methods, the similarity calculation is based on a structural image instead of PET (Figure 2). The proposed CONN-NLM filter combines the NLM similarity weighting in Eq. (2) with the hybrid connectivity measure in Eq. (4) to form a connectome-based weighting:


(5) w′i⁢j=wi⁢j×Ai⁢j=1Z′⁢(i)×e⁢x⁢p⁢(-||xi→-xj→||2,a2h2)×Ai⁢j

*w′_*i,j*_* is normalised similarly to Eq. (3), by dividing by *Z(i)* where:


(6) $$Z^{\prime }\,\left(i\right)=\sum_{j}e\,x\,p\,\left(-\frac{{||\vec{x_{\text{i}}}-\vec{x_{\text{j}}}||}_{2,a}^{2}}{h^{2}}\right)\times A_{i\,j}$$

Applying this combined weight with the CONN-NLM filter, the filtered PET intensity value at voxel *i* becomes:


(7) C⁢O⁢N⁢N⁢_⁢N⁢L⁢(xi)=∑j∈N⁢(i)w′i⁢j×xj=∑j∈N⁢(i)1Z′⁢(i)×e⁢x⁢p⁢(-||xi→-xj→||2,a2h2)×Ai⁢j

**FIGURE 3:**
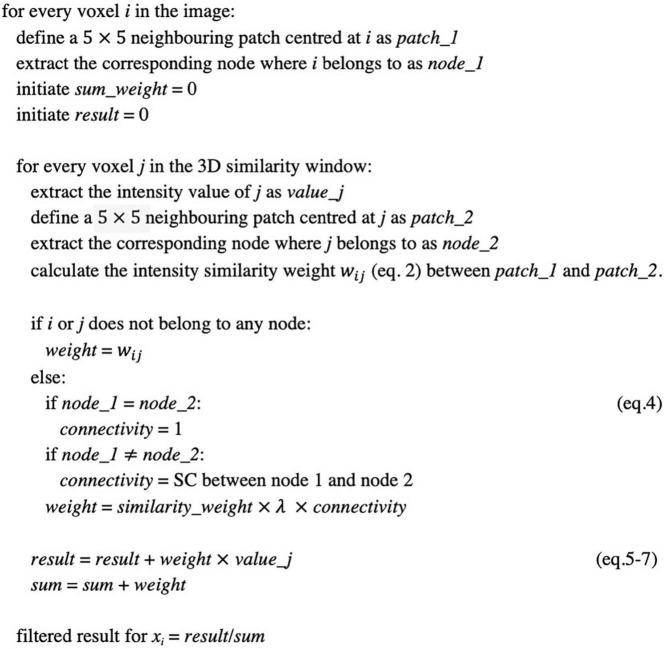
Pseudocode for implementing the connectome-based non-local means (CONN-NLM) filter.

Figure 3 provides a step-by-step pseudo-code for implementing the CONN-NLM filter. Because tractography measures 3D structural information, the CONN-NLM filter is extended to perform 3D smoothing similar to the approach used in the method proposed by Chan et al. (2014). This 3D smoothing method computes the similarity between 2D patches in a 3D search window. In the current study, the 3D search window *N*×*N*×*S* (S represents the number of slices) is defined as the entire image volume, and the size of the 2D patches, *M*×*M*, is defined as 5×5.

### Parameter Estimation

#### Similarity Smoothing Strength (h)

The parameter *h* in Eq. (2) regulates the NLM filter strength and, following recommendations by Chan et al. (2014) is set proportional to the PET image noise level:


(8) $$h^{2}=C\times \sigma_{P\,E\,T}^{2}$$

where $\sigma_{P\,E\,T}^{2}$ is the standard deviation of PET voxel values and *C* is a constant (Chan et al., 2014). In general, the noisier the PET data prior to filtering, the larger the filtering strength. Users can fine-tune *h*^2^ to achieve a desired smoothness outcome.

#### Connectivity Ratio (λ)

Similar to the NLM filter strength, and to scale in proportion to the connectivity data quality, the parameter λ in Eq. (4), which corresponds to the ratio of distant-to-local connectivity, can be set to be proportional to the variance of the track-density image (TDI) (Calamante et al., 2010):


(9) $$\lambda =B\times \sigma_{T\,D\,I}^{2}$$

where B is a constant regulating the distant-to-local connectivity weighting to account for the connectome sensitivity and tracking quality: the worse the quality of the diffusion MRI data, the worse the quality of the fibre-tracking results (and thus the lower the reliability of the distant connectivity estimates), which translates to lower contrast of the associated TDI map (Calamante et al., 2010); this in turn results in a lower λ value, consistent with less weighting given to the distant connectivity term in the case of lower diffusion MRI quality data. A number of factors affect TDI variance including initial diffusion weighted imaging quality, tractography processing methods, and TDI resolution (Calamante, 2017). Depending on specific applications, fine tuning can be performed to find the optimal connectivity ratio – see below for an example.

## Experiments and Evaluation

### Simulated Data

**FIGURE 4:**
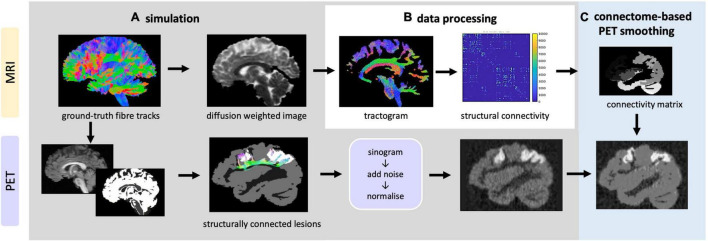
Overview of data simulation, processing and CONN-NLM filtering framework. **(A)** Top: simulation of DWIs based on ground-truth fibre tracks; bottom: simulation of raw PET sinograms, which are then reconstructed to generate PET images **(B)** the simulated DWIs are processed to generate tractogram and structural connectivity matrices **(C)** the proposed post-reconstruction filter is applied to improve the PET image quality.

In order to test and optimise CONN-NLM, a novel PET/dMRI simulation framework (Figure 4) was developed to simulate realistic PET/dMRI data. Detailed instructions to generate the phantom are included in the Supplementary Material.

#### Diffusion Simulation

A realistic brain diffusion-weighted image (DWI) dataset (2 mm isotropic, 90 diffusion-encoding directions, b = 1,000, 2,000, 3,000 s/mm^2^, similar to the diffusion gradient scheme used in the Human Connectome Project) was simulated based on 25 manually segmented white matter fibre bundles from the ISMRM 2015 Tractography challenge data (Maier-Hein et al., 2017) using the Fiberfox (Neher et al., 2014) software. The simulated multi-shell DWI was up-sampled to 1 mm^3^ resolution for tractography processing.

#### Positron Emission Tomography Simulation

**FIGURE 5:**
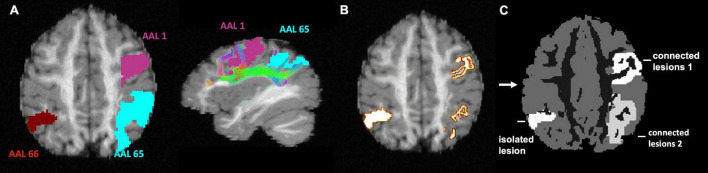
Lesion phantom simulation. **(A)** AAL1 and AAL65 are highly connected regions; ALL66 is not connected to either AAL1 or AAL65. **(B)** The endpoints of ground-truth white matter tracks connecting AAL1 and AAL65 regions are extracted. The entire AAL66 region is used to create an isolated lesion. **(C)** The selected endpoints in AAL1 and AAL65 were dilated to form uniform high-intensity lesions, which were added to segmented T1 data to form a phantom image for PET simulation. This image was also used as the ground-truth for method evaluation.

Analytical PET simulation was performed based on a modified T1-weighted image with added simulated lesions. The T1-weighted structural image is a fabricated T1-weighted image based on the 25 white matter fibre bundles used in diffusion simulation (Maier-Hein et al., 2017). The T1-weighted image was first segmented into grey matter (GM), white matter (WM), and CSF tissue types using Volbrain (Manjón and Coupé, 2016). Because the proposed CONN-NLM filter uses network connectivity information, both connected and isolated lesions are needed to properly evaluate the effects of the filter. To simulate lesion phantoms, the endpoints of specific ground-truth fibre tracks were first selected as the starting seeds. While the choice of lesion locations can be arbitrary in a simulated phantom, we placed the simulated lesions within the dorsal attention network (DAN), one of the major white matter pathways affected in Alzheimer’s disease (Hansson et al., 2017). The starting seeding points were filtered to be exclusively located within three major nodes within the DAN approximately in the AAL1, AAL65, and AAL66 nodes. The fibre endpoints of the ground-truth fibre tracks in AAL1 and AAL65 were extracted and dilated to form clusters of voxels to represent two lesion areas (Figure 5). AAL1 and AAL65 have strong ipsilateral connections, while there is no contralateral connection to AAL66. Thus, the entire AAL66 was extracted to form an isolated lesion. Note that for the two connected lesions, the seeding voxels were chosen within a particular ROI, but after dilation, the lesions were not constrained within a single AAL region. To avoid confusion, the lesions with seeding voxels in AAL1, AAL65, and AAL66 were named as “connected lesion 1,” “connected lesion 2,” and “isolated lesion,” respectively. In this way, a realistic brain simulation was constructed, containing three lesions (two interconnected and one disconnected), thus allowing to investigate the performance of the method under a range of connectivity conditions.

The ground-truth PET phantom image was generated by assigning a uniform value to each segmented class. In particular, lesions were simulated with two intensity levels, approximately × 2.5 (for the lesions in AAL1 and AAL66) or × 1.8 (for the lesion in AAL65) higher than GM intensity. Noisy PET sinograms were simulated in ASIM (Elston et al., 2012) based on the phantom image with a total count of 10^7^ and 10^8^ at 20% noise. The simulated PET sinograms were normalised and reconstructed using OSMAP-OSL within the STIR package (Thielemans et al., 2012) for a total of 60 sub-iterations (12 subsets).

### Clinical Data

Three sets of clinical data containing tau-PET, dMRI, and T1w images were obtained from the Alzheimer’s Disease Neuroimaging Initiative (ADNI) database (adni.loni.usc.edu) to illustrate the proposed CONN-NLM filter on a real patient example.

The T1-weighted scan was acquired at 2 × 2 × 2 mm^3^ resolution. dMRI was acquired using the ADNI3 advanced protocol, with b = 500, 1,000, 2,000 s/mm^2^ applied in a total 112 diffusion weighted directions, and with voxel size: 2 × 2 × 2 mm^3^. The tau-PET images were acquired using the standard ADNI3 AV-1451 PET protocol with 300 MBq ± 10% injected, and six 5-min frames were acquired. Please refer to ADNI3 Procedures Manual Version 3.0 for more details regarding the acquisition protocol and patient inclusions/exclusion criteria. Our research included MRI and PET images of a healthy subject, a mild cognitive impaired (MCI) patient, and a Alzheimer’s patient. All three subjects met all of our selection criteria, including undergoing both dMRI and PET within 15 days, and having dMRI with advanced protocols. Both the PET and T1-weighted images were co-registered to the diffusion space using the FSL registration tool (Jenkinson et al., 2002).

### Fibre-Tracking Processing

Tractography analysis was performed using MRtrix software (Tournier et al., 2012, 2019). Multi-shell multi-tissue constrained spherical deconvolution (Jeurissen et al., 2014) was used to compute the local fibre orientation distributions, and probabilistic diffusion tractography (10 million tracks) was carried out using Anatomically Constrained Tractography (ACT) framework (Smith et al., 2012), followed by spherical deconvolution informed filtering of tractogram 2 (SIFT2) (Smith et al., 2015), which was applied to optimise the connectivity quantification accuracy. To perform quantitative connectome analysis, the AAL brain atlas (Rolls et al., 2020) (170 node parcellation) was chosen to construct a SC matrix, based on the sum of the SIFT2 weights for the streamlines connecting each pair of nodes. For the clinical patient dataset, the extra following pre-processing steps were also carried out: the DW images were first corrected for noise and bias-field, and distortion correction was performed using Synb0 (Schilling et al., 2019).

### Quantitative Evaluations

In addition to visual inspection, quantitative evaluations of the entire image and lesion ROIs were performed to assess the effect of the CONN-NLM filter on image quality. The following quantitative metrics were used (Chan et al., 2014):

#### Mean Squared Error

Mean Squared Error (MSE) is defined as:


(10) $$M\,S\,E=\frac{1}{N}\,\sum_{j}{\left(x_{j}^{T\,R\,U\,E}-x_{j}\right)}^{2}$$

where *N* is the number of voxels in the entire image, *x*_*j*_is the intensity value of filtered PET image, and $x_{j}^{T\,R\,U\,E}$is the intensity value of the ground-truth PET phantom image.

#### Lesion Contrast-to-Noise Ratio

Contrast-to-Noise Ratio (CNR) is defined as:


(11) $$C\,N\,R=\frac{M_{L}-M_{G\,M}}{\sigma_{G\,M}}$$

where *M_L_* is the median lesion intensity and *M*_*GM*_ is the median background intensity measured in the GM region. The median was used because the ROI mask covered the entire lesion region whose values have a bimodal distribution. Using the mean in a non-normal distribution might lead to significant quantification bias. Noise was measured by σ_*GM*_, the variance of voxel values in the GM. In order to examine lesions with different connectivity characteristics, lesion CNRs were computed for each of the three lesions separately.

#### Lesion Contrast Recovery Coefficient

Contrast Recovery Coefficient (CRC) is defined as:


(12) $$CRC\left(\%\right)=\frac{\frac{M_{L}}{M_{G\,M}}-1}{C\,R-1}\times 100$$

where CR is the true lesion-to-background contrast, *M_L_* is the measured median lesion intensity, and *M*_*GM*_ is the median background intensity measured in normal GM.

## Results

### Simulated Data

#### Optimisation of Filter Parameters

We recommend using the following guidelines to find initial estimates of *h*^2^ and λ, followed by fine-tuning in specific applications. As initial estimates, we used *C* = 8 for the simulated phantom to find a range for testing. According to equation 8, *h*^2^ was estimated to be 0.18 with a background noise variance of 0.023. We further expanded the range for testing to *h*^2^ = 0.05 – 0.3 to better understand the effect of filtering strength *h*^2^ on performance. As for the connectivity ratio λ, we used *B* = 0.5×10^−5^for the artefact-free, high-quality TDI, corresponding to λ = 1. Based on this initial estimate, we expanded the test range to λ = 0 – 10^3^.

**FIGURE 6:**
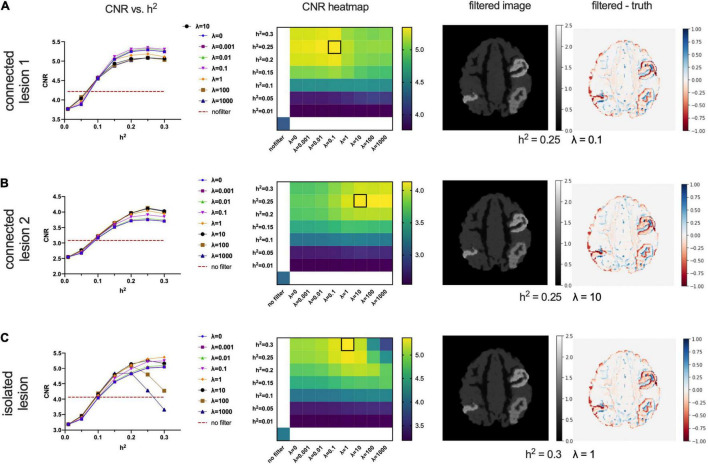
Optimising the filtering strength (*h*^2^) and connectivity ratio (λ) according to lesion contrast-to-noise ratios for connected lesion 1 (AAL1), connected lesion 2 (AAL65), and the isolated lesion (AAL66) in the high-count phantom. The first column shows CNR vs. *h*^2^ plots at different λ values, where λ = 0 means only intra-node voxels are included to calculate similarity weights. The horizontal dashed lines indicate the baselined CNR for the noisy PET image without filtering. The second column shows CNR heatmaps; the location of the brightest colour (outlined with a black box) in the CNR heatmaps indicates the preferred *h*^2^ (y-axis) and λ values (X-axis). The last two columns present the filtered images and the corresponding difference maps (filtered – ground truth image). The chosen parameters are *h*^2^ = 0.25,λ = 0.1in panel **(A)**, which is optimal for the connected lesion in AAL1; *h*^2^ = 0.25,λ = 10 in panel **(B)**, which is optimal for the connected lesion in AAL65; and *h*^2^ = 0.3,λ = 1 in panel **(C)**, which is optimal for the isolated lesion in AAL66.

Figure 6 shows the performance of the proposed CONN-NLM filter as a function of the filtering parameters, *h*^2^ and λ, for the high-count simulated PET phantom. CNRs are plotted for each of the three lesions separately, as they differ in their inter-regional connection (high structural connection vs. no connection) and intensity values (connected lesions have different intensity). Connected lesion 1 refers to the lesion in AAL1 (upper right location); connected lesion 2 refers to the lesion in AAL65 (lower right); and the isolated lesion is located in the lower left of the image slice in AAL66. The curves for λ = 0 refer to the special case when smoothing is based only on voxels within the same node, or local smoothing. At larger values of λ, the weighting of distant connectivity increases. Higher CNRs at λ > 0 suggest improvements made by introducing information about distant structural connectivity. In broad terms, CNR increases as *h*^2^ increases for the connected lesions 1 and 2. For the isolated lesion, however, as *h*^2^ reaches large values (>0.2), CNR decreases for λ= 100 and λ= 1,000. When considered across connectivity ratios, the best CNR occurs at λ= 0.1, 10, and 1 for the connected lesions 1 and 2, and the isolated lesion, respectively. The highest lesion CNR is achieved for connected lesion 1 when *h*^2^ = 0.25 and λ= 0.1; for connected lesion 2 when *h*^2^ = 0.25 and λ= 10; and for isolated lesion 1 when *h*^2^ = 0.3 and λ= 1. The optimal parameters are highlighted by square boxes in the CNR heatmaps to indicate the highest CNRs (Figure 6). Based on the combined results of all three lesions, the optimal overall performance is achieved when *h*^2^ = 0.25 and λ = 1.

The corresponding results for the low count simulation are shown in Supplementary Figure 1. Using the same constants as the high-count simulation, *C* = 8,*B* = 0.5×10^−5^, the initial estimates were *h*^2^ = 0.44,λ = 1. Overall, the low count results showed similar trends, with CNR increasing as *h*^2^ increases for connected lesions 1 and 2. The optimised parameters for all three lesions are similar to each other. In both simulations, Because the low count simulation is noisier, higher *h*^2^value at 0.4 is expected [i.e., see Eq. (8)]. The CNR curve of the isolated lesion is slightly different from the high-count simulation where there is an initial decrease in CNR for high λ.

#### Comparison With Other Filtering Methods

**FIGURE 7:**
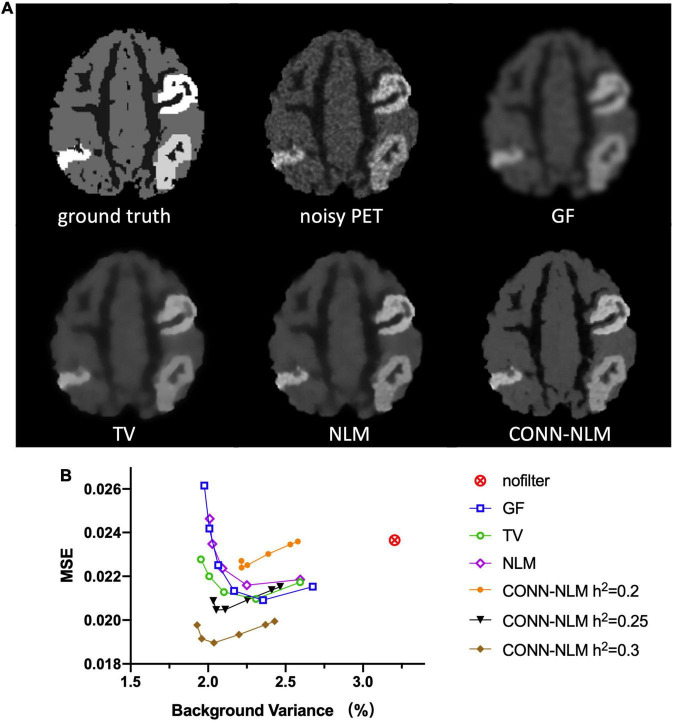
Denoising effects on the high-count simulated PET data. **(A)** From left to right and from top to bottom: ground truth noiseless image, unfiltered noisy PET image, Gaussian filtering (GF), total variation (TV), non-local means (NML) filtering, and the proposed CONN-NLM filtering. **(B)** Mean squared error (MSE) for the whole image plotted against the normal GM noise variance (%). For each filter, six points were chosen to produce approximately the same range of noise variance, by varying the corresponding filter parameter.

For comparison purposes, Figure 7A shows the images filtered with a Gaussian filter (GF), a total variation filter (TV), the nonlocal means (NLM) filter, and the proposed CONN-NLM filter. While GF reduced noise significantly, it also blurred tissue boundaries and lesions which could lead to quantitative bias due to partial volume averaging. Both TV and NLM filters reduced the overall noise and improved lesion contrast, with NLM significantly improving the contrast of connected lesion 1 (upper right lesion). CONN-NLM further improved the image by increasing the contrast of the lesions, and by sharpening the image across tissue boundaries. Figure 7B displays the quantitative evaluation of the filtered images in Figure 7A by computing the overall image MSE vs. background noise variance (%). For each filter, six points were chosen to produce approximately the same range of noise variance, by varying the corresponding filter parameter. The six points on the CONN-NLM correspond to connectivity ratios of λ = 0, 0.01,1,10,100 (from right to left). Additionally, three filtering levels at *h*^2^ = 0.2, 0.25, and 0.3 were plotted for CONN-NLM. CONN-NLM with *h*^2^ = 0.25 and *h*^2^ = 0.3 outperformed the GF, NLM, and NLM filters by producing the lowest MSE at comparable noise variance.

**FIGURE 8:**
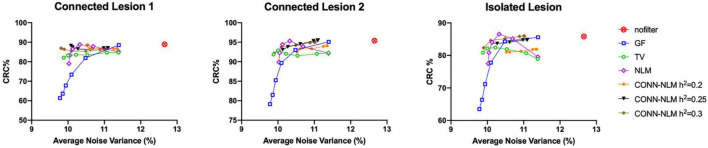
CRC% plotted against the average variance (background and lesion variance combined) for each lesion separately, for the high-count simulated PET data. For each filter, six points were chosen to produce approximately the same range of noise variance, by varying the corresponding filter parameter.

For each lesion, Figure 8 plots the lesion CRC vs. average noise variance. Average variance is calculated by combining variances of the normal GM region and the indicated lesion, i.e., (σ^2^_*GM*_ + σ^2^_*lesion*_)/2, to reflect the effect of the filters on performance in both areas. In the noisy (unfiltered) PET image, the CRC is very high, but the variance is also very high because of the noise. GF and NLM reduce noise at the cost of reduced lesion contrast. NLM produced the highest CRC. However, when the variance continues to decrease, the CRC also drops rapidly because the lesions have been oversmoothed at higher filtering strengths. Both TV and CONN-NLM filters reduce noise without compromising the lesion CRCs, as indicated by their flat response curves. CONN-NLM performs slightly better than TV filter with higher overall CRC.

The low count simulation follows a very similar performance (Supplementary Figure 2). CONN-NLM with *h*^2^ = 0.3, 0.4, and 0.5 outperforms GF, TV, and NLM filtering.

### Clinical Data

**FIGURE 9:**
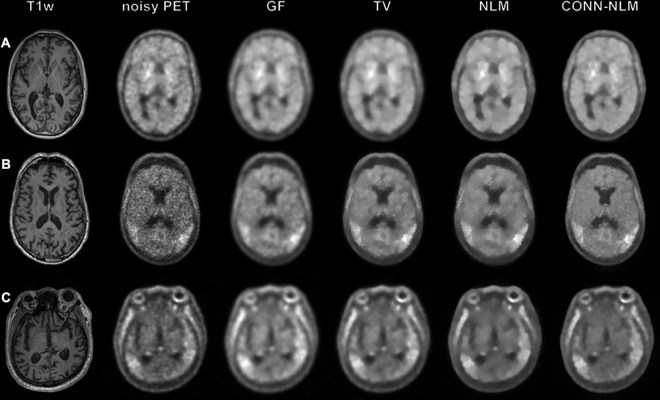
Applying image smoothing on clinical tau-PET and MRI data from **(A)** a health-control subject **(B)** a mild cognitive impairment patient and **(C)** a Alzheimer’s disease patient. From left to right: T1-weighted image, noisy PET image, Gaussian filtering (GF), total variation (TV), nonlocal means (NLM) and the proposed CONN-NLM filtering. Filtering parameters were chosen for all filters to produce approximately the same level of image noise.

Figure 9 shows the filtered results from three clinical dataset from the ADNI3 study. These three examples demonstrate three different diagnostic groups including a health control, a MCI patient, and an Alzheimer’s patient. In contrast to the simulated data, it should be noted that the noisy PET image has been already post-smoothed according to the standard ADNI3 processing protocol, and no unfiltered data were available in this case. We compared the post-filtering results using GF, TV, NLM, and CONN-NLM filters. We chose *h*^2^ = 2300 (C = 8, σ^2^ = 6.7×10^8^) as an initial estimate and fine-tuned the parameters. For comparison purposes, parameters were chosen to produce approximately the same level of noise for each filter. CONN-NLM was implemented with *h*^2^ = 200 and λ = 1, *h*^2^ = 3,000, and λ = 1, *h*^2^ = 2,000, and λ = 1. GF suppressed noise but also blurred tissue boundaries. TV suppressed noise, similar to GF, and yielded slightly sharper boundaries between the regions of enhanced tau-PET signal and normal grey matter. Both NLM and CONN-NLM further improved the images by suppressing noise in the GM. CONN-NLM showed higher contrast in regions of enhanced tau-PET signal and clearer boundaries between tissues than the other methods.

## Discussion

This study proposed CONN-NLM, a new method of denoising PET images taking into account structural connectivity information from dMRI data from the same subject. The method assumes that structurally connected voxels (i.e., the “CONN” part of the method) and voxels with similar intensity patterns (i.e., the NLM part of the method) should be highly weighted when performing non-local denoising. CONN-NLM effectively brings further non-local information into the denoising process. CONN-NLM exploits the highly associated information that can be present in dMRI and PET data (McAleese et al., 2017; Jacobs et al., 2018; Mito et al., 2018; Wen et al., 2021), to provide a new means to synergise the structural connectivity from dMRI and the molecular imaging information from PET. Specifically, the proposed filter smooths across grey matter regions that are structurally connected via white matter tracts. The method exploits the information from the endpoints of the diffusion MRI streamlines, and therefore, relates to the PET signals arising from the grey matter areas whose white matter pathways are connected. We have demonstrated that this method effectively suppresses noise while preserving or improving lesion contrast in both simulated and clinical data.

### Data Simulation

As there was no appropriate simulated data that realistically simulated the brain structural connectivity and the PET lesions, we developed here a new simulation framework (Figure 3) to generate realistic hybrid dMRI and PET data in order to evaluate the methods with reference to a known ground-truth. dMRI data were simulated using manually segmented white matter tracts, with a brain phantom that is currently considered among the most complex and realistic (Maier-Hein et al., 2017). Raw PET data were simulated using the modified structural T1-w image as the voxelised phantom and a realistic PET simulation method (Elston et al., 2012). Our combined simulation framework not only provides perfectly overlapping dMRI and PET images without requiring registration but importantly provides flexibility to add lesions with specific user-defined locations, anatomical characteristics, PET contrast, as well as connectivity information for particular applications.

A similar concept of hybrid connectivity has been applied in a different context, to smooth EEG signals (Hammond et al., 2013). In that study, a distance-based local connectivity and a dMRI-based structural connectivity based distant connectivity were combined to form a hybrid connectivity to effectively smooth EEG signals. Similarity, we use the parameter λ to control the ratio of distant to local connectivity, with λ = 0 indicating the weighting is based only on voxels within the same node. The method we propose is the first attempt to include information from distant nodes for the denoising of PET data, which is especially useful when lesions are connected via white matter pathways. When selecting an appropriate λ ratio for filtering, it is vital to take into consideration the quality of dMRI data when determining the accuracy of connectivity inferences (see “Estimating Parameters” section below).

In the current study, we used the AAL atlas with 180 nodes in both the simulated and clinical data. However, we expect different atlases can be used for specific applications. With increased parcellation resolution, a higher number of streamlines should be generated in order to construct a reliable connectome. Users can also choose a network-based connectome to explore regions of interest within particular networks.

### Lesion Characteristics

The distribution of lesions in neuropathological diseases is extremely diverse, and a thorough investigation of the range of configurations is beyond the scope of this study. We, however, created three lesions with different connectivity characteristics to test the effect of the proposed filtering method under varying conditions. Indeed, the filter affected the isolated lesions differently than the connected lesions (Figure 6), especially at higher filtering strengths (*h*^2^ > 0.25) and high weighting of distant connectivity (λ > 100). This behaviour is due to the lack of underlying white matter bundles in node AAL66 that connect to nodes AAL1 and AAL65, where the two other lesions were located. By increasing λ, more weight is given to structural connectivity information, which might not be as relevant for isolated lesions. However, when local and distant connectivity is balanced, such a method can still provide accurate smoothing. Using different contrast levels in the connected lesions, we assessed the robustness of the proposed method. Figure 6 shows that the CONN-NLM filter can handle various lesion contrasts consistently. While the optimal parameters can vary across lesions, CNRs are only impacted slightly. Choosing one set of optimal parameters does not result in significant compromises on individual lesion CNR. By introducing information in distant voxels when λ > 0, the proposed method does not adversely affect the unconnected lesions, making the filtering effects robust to the choice of filter parameters.

### Estimating Parameters

Connectome-based non-local means filtering uses two user-defined parameters, filtering strength (*h*^2^) and connectivity ratio (λ). We optimised these parameters for the simulated data to understand the behaviour of the algorithm and compare them with other filtering methods. Optimising parameters conclusively is impossible with clinical data that lacks any ground truth. Based on the variance of the noise on the PET data and the quality of the TDI maps from dMRI, we provide guidelines for choosing these parameters (Eqs. 8, 9). Similarly to the original NLM formula, *h*^2^ defines the amount of Gaussian weighting used in the similarity calculation (Buades et al., 2005). When images are noisier, higher *h*^2^ values produce stronger filter effects. By expressing the filtering parameters as a function of the noise variance in the PET and TDI data (Eqs. 8, 9), it allows to reduce the dependency of the filter on image quality, and represent them in terms of the constants B and C, which should be less sensitive to image noise. For example, while *h*^2^ was different for the high-count and low-count cases, they led to an approximately constant C value.

### Potential Applications

Brain lesions not only result in local anatomical abnormalities, but they can be also highly related to structural, functional, and cognitive behaviour at the network level (Thiebaut de Schotten et al., 2020). While the CONN-NLM method is a connectome-based filtering method, it inherently contains anatomical information when constructing the structural connectivity matrix. Based on the premise that radiotracer uptake is relatively more homogeneous within tissue boundaries, the nonlocal means component can provide informative noise reduction while preserving edge information. The brain parcellation can be also tailored to meet specific needs, for example, by creating disease-specific nodes.

Tau-PET data and dMRI data in Alzheimer’s disease were used to demonstrate the effectiveness of this method since there is a strong correlation between tau distribution and WM pathways (McAleese et al., 2017; Jacobs et al., 2018; Mito et al., 2018; Bischof et al., 2019; Wen et al., 2021). The white matter bundles in the brain might connect lesions in distant parts of the brain, thus introducing extra connectivity-informed contributions to the non-local PET filtering. Other potential applications include but are not limited to neurodegenerative diseases (Aiello et al., 2019) and oncology (Sotoudeh et al., 2016). For applications where established links exhibit, this methodology can be applied exploit the synergy between PET tracer distribution and structural connectivity network. However, the benefits of this method will not be realised in applications where changes in the PET signal and WM pathways do not exhibit correlations.

### Limitations

The CONN-NLM filtering resulted in a decreased CNR compared to the noisy PET image at low filtering strengths (*h*^2^ < 0.1 in Figure 6). Our method differs from most of the other local smoothing and NLM smoothing methods since we include a larger window size. The weighting needs to be more informative when weighting across a large number of voxels. An extremely low number of *h*^2^ is equivalent to averaging all voxels in an image. Therefore, we recommend not using a very weak filtering strength when performing CONN-NLM filtering.

While the CONN-NLM filter was shown to improve PET image quality in a number of simulated scenarios, it is not possible to cover all cases of lesions and network characteristics in one study. Especially in images where no distinct lesion is present, the CONN-NLM filter might not have a distinct advantage in comparison to other anatomically informed filtering methods. It should be noted, however, that when the parameters are properly chosen, the method does not penalise focal disconnected lesions; in these lesions, local smoothing within the node is still applied.

The accuracy of the smoothing will depend on the quality of the structural connectivity information. Previous studies have demonstrated that FODs and tractography can be computed in the presence of abnormalities in clinical studies (Dhollander et al., 2021). Errors during tractography will likely propagate to subsequent analysis including connectomes and the connectome-based filtering. To minimise this source of error, in this study we used state of the art tractography methods, which included (Calamante, 2019): dMRI model for fibre orientations that are robust to crossing fibres (Tournier et al., 2007), probabilistic tractography algorithm (Tournier et al., 2012) with anatomical constrains (Smith et al., 2012) and streamline filtering for tractography quantification (Smith et al., 2015). Furthermore, we provided guidelines in the parameter estimation section. The parameter *C* is determined based on TDI contrast, which in turn depends on the quality of tractography.

Besides the obvious lack of ground-truth information, there were a number of other limitations regarding our *in vivo* example. First, we had to co-register the MR and PET clinical images, as the data were not acquired simultaneously on a hybrid PET/MR system. Second, CONN-NLM should ideally be applied to unfiltered PET data; the available ADNI data were, however, heavily smoothed. Finally, the accuracy of smoothing can be affected by the dMRI acquisition protocol; this may impact the performance of CONN-NLM when applied to retrospective data acquired with dMRI protocols optimised for Diffusion Tensor Imaging.

Non-local means filters typically employ a 13 × 13 similarity window (Chan et al., 2014). Our methods, however, expand the similarity window to include the entire image, which can require a lot of computational resources. As a reference, processing a typical 126-slice 3D PET volume using a 32G memory, 3.2 GHz *12 Core CPU local system takes about 4–5 h. It may be helpful to implement a faster version of the NLM algorithm to increase the speed of the filter. For example, this algorithm can be highly parallelised (e.g., processing individual voxels in parallel), which has the capacity to speed up with increasing number of threads. Furthermore, the current results were obtained based on a simple Python code implementation, which leaves great scope for improvements in code efficiency and computational time.

## Conclusion

Our simulated phantom and clinical data demonstrate that the proposed CONN-NLM method effectively suppresses PET noise while maintaining lesion contrast. By incorporating structural connectivity information into the non-local smoothing of the PET data, lesion CNRs can be improved. CONN-NLM filtered PET images showed better edge preservation and lower noise than the same images filtered with other local and non-local filtering methods. In summary, we showed that combining information from dMRI and PET during image postprocessing provides a new avenue for multimodal image synthesis.

## Data Availability Statement

The original contributions presented in the study are included in the article/Supplementary Material, further inquiries can be directed to the corresponding author.

## Author Contributions

ZS performed the method implementation and data analysis and wrote first draft of the manuscript. All authors contributed to the conception and design of the study, as well as the interpretation of findings, manuscript revision, and approved the submitted version.

## Conflict of Interest

The authors declare that the research was conducted in the absence of any commercial or financial relationships that could be construed as a potential conflict of interest.

## Publisher’s Note

All claims expressed in this article are solely those of the authors and do not necessarily represent those of their affiliated organizations, or those of the publisher, the editors and the reviewers. Any product that may be evaluated in this article, or claim that may be made by its manufacturer, is not guaranteed or endorsed by the publisher.

## Footnotes

1 A preliminary version of this work was presented at the 29th Annual Meeting of the International Society for Magnetic Resonance in Medicine (Sun et al., 2021).

2 Data used in preparation of this article were obtained from the Alzheimer’s Disease Neuroimaging Initiative (ADNI) database (adni.loni.usc.edu). As such, the investigators within the ADNI contributed to the design and implementation of ADNI and/or provided data but did not participate in analysis or writing of this report. A complete listing of ADNI investigators can be found at: http://adni.loni.usc.edu/wpcontent/uploads/how_to_apply/ADNI_Acknowledgement_List.pdf.

3
https://www.mrtrix.org/
