# Supplementary material for: CONN-NLM: A Novel CONNectome-Based Non-local Means Filter for PET-MRI Denoising
Source: Front Neurosci. 2022 May 30;16:824431. doi: 10.3389/fnins.2022.824431 (PMC9197079; doi:10.3389/fnins.2022.824431)
Supplement: Supplementary file 1 [file Image_1.PDF]

Supplemental figures

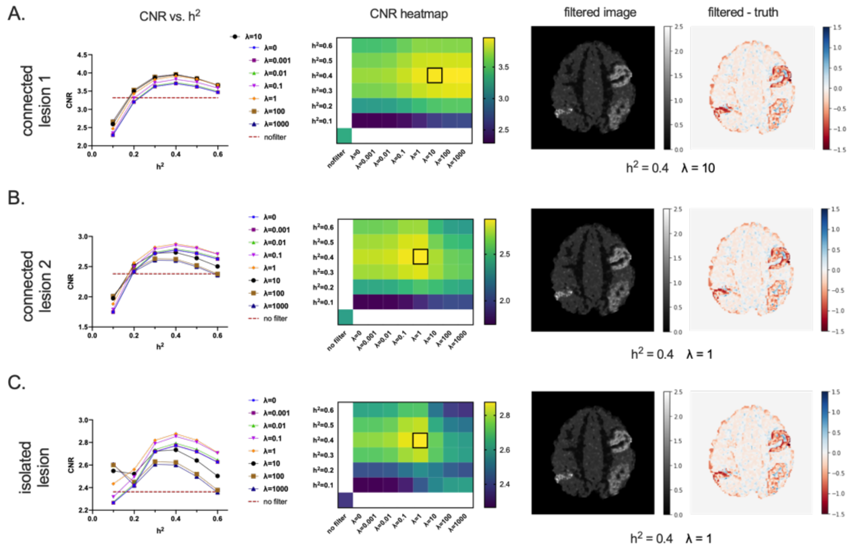

S1. Optimising the filtering strength (h^2) and connectivity ratio (λ) according to lesion contrast-to-noise ratios for connected lesion 1 (AAL1), connected lesion 2 (AAL65), and the isolated lesion (AAL66) in the low-count phantom. The first column shows the CNR vs. h^2 plots at different λ values, where $\lambda = 0$ means only intra-node voxels are included to calculate similarity weights. The horizontal dashed lines indicate the baseline CNR for the noisy PET image without filtering. The second column shows CNR heatmaps; the location of the brightest colour (outlined with a black box) in the CNR heatmaps indicates the preferred h^2 (y-axis) and λ values (x-axis). The last two columns present the filtered images and the corresponding difference maps (filtered – ground truth image). The chosen parameters are $h^2 = 0.4, \lambda = 10$ in (**A**), which is optimal for the connected lesion in AAL1; $h^2 = 0.4, \lambda = 1$ in (**B**), which is optimal for the connected lesion in AAL65; and $h^2 = 0.4, \lambda = 1$ in (**C**), which is optimal for the isolated lesion in AAL66.

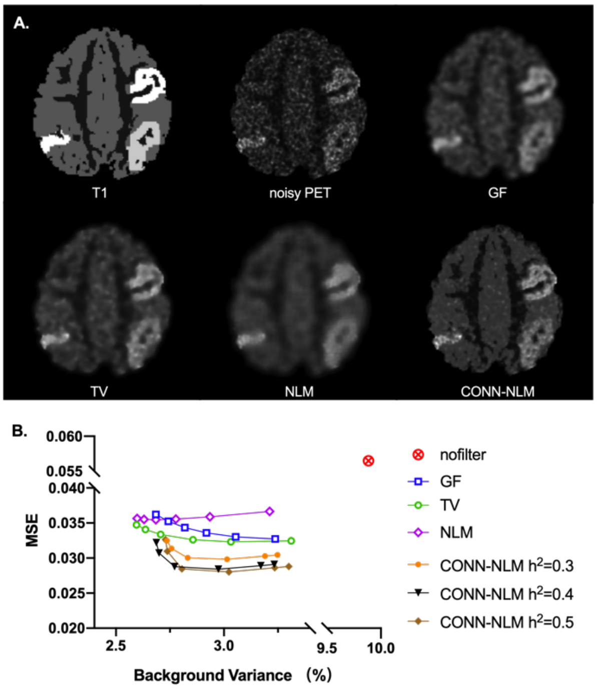

S2. Denoising effects on the low count simulated PET data. (**A**) From left to right and from top to bottom: ground truth noiseless image, unfiltered noisy PET image, Gaussian filtering (GF), total variation (TV), non-local means (NML) filtering, and the proposed CONN-NLM filtering. (**B**) Mean squared errors (MSE) for the whole image plotted against the normal GM noise variance (%). For each filter, six points were chosen to produce approximately the same range of noise variance, by varying the corresponding parameter of relevance.
