# Supplementary material for: CONN-NLM: A Novel CONNectome-Based Non-local Means Filter for PET-MRI Denoising
Source: Front Neurosci. 2022 May 30;16:824431. doi: 10.3389/fnins.2022.824431 (PMC9197079; doi:10.3389/fnins.2022.824431)
Supplement: Supplementary file 2 [file Presentation_1.pdf]

Simulation documentation (print)

Required software

simulation tools

- FibreFox DW-MRI Simulation: http://docs.mitk.org/2016.11/org_mitk_views_fiberfoxview.html
- ASIM: https://depts.washington.edu/asimuw/

image reconstruction

- STIR: http://stir.sourceforge.net/

image processing

- MRtrix3: https://www.mrtrix.org/
- volBrain: https://volbrain.upv.es/

data visualisation and processing

MATLAB, ImageJ, Python Jupyter-notebook

Data for simulation

download data from **ISMRM 2015 Tractography challenge:** http://tractometer.org/ismrm_2015_challenge/

- basic dataset (contain T1 file)
- ground truth bundle (fibers.fib)

Diffusion data simulation

- only noise artefact
- **DWI** [90 * 108 * 90 * 288   2mm^3]

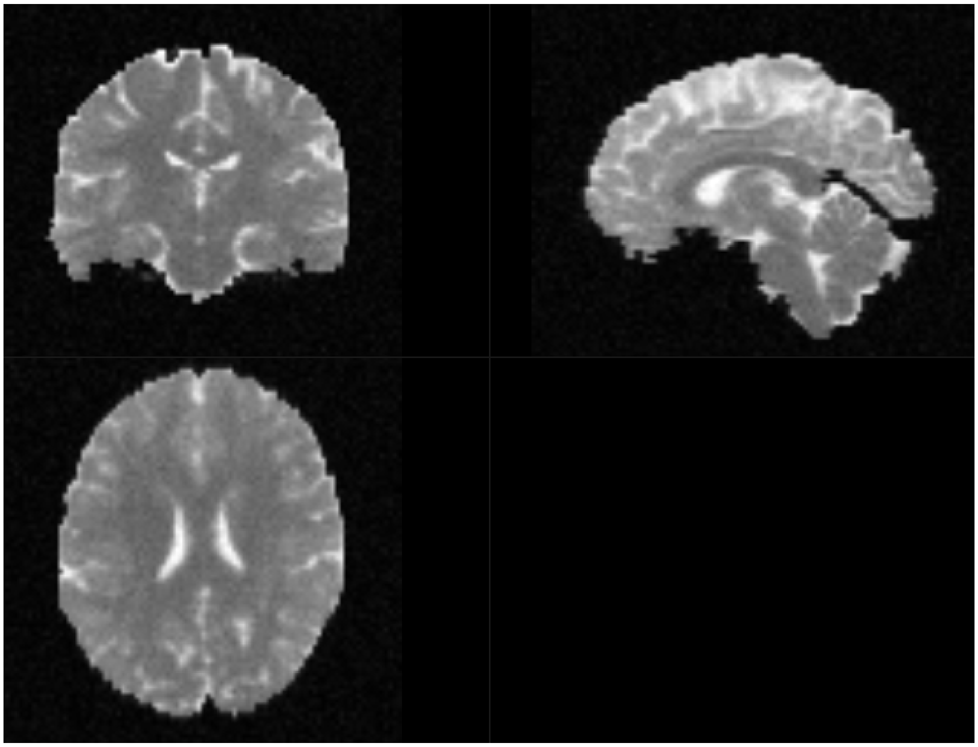

```
PLEX-GAUSSIAN-251.log
0:0:0 > Starting Fiberfox dMRI simulation
Output image spacing: [2,2,2]
Output image size: [90,108,90]
Working image spacing: [2,2,2]
Working image size: [90,108,90]
Using tissue mask
Using volume fraction map for fiber compartment 1
Using volume fraction map for fiber compartment 2
Using volume fraction map for non-fiber compartment 1
Using volume fraction map for non-fiber compartment 2

Setting fiber radius to 18.14µm to obtain full voxel.

0:0:9 > Generating 4-compartment diffusion-weighted signal.
b-values: 5 1000 1995 3005 995 2995 2005 990 1990 3000 1985 2990 1005 2000 3010 2985 2010 2994 2980
Volumes: 288

0% 10 20 30 40 50 60 70 80 90 100%
|----|----|----|----|----|----|----|----|----|----|

---

0:6:47 > Simulating k-space acquisition using 1 coil(s)
Acquisition type: single shot EPI
Simulating signal relaxation
Simulating complex Gaussian noise: 251
Parallel volumes: 4
Threads per slice: 3
0% 10 20 30 40 50 60 70 80 90 100%
|----|----|----|----|----|----|----|----|----|----|

---

8:40:55 > Finalizing image
0% 10 20 30 40 50 60 70 80 90 100%
|----|----|----|----|----|----|----|----|----|----|
```

```
---

8:40:55 > Finished simulation
```

PET data simulation

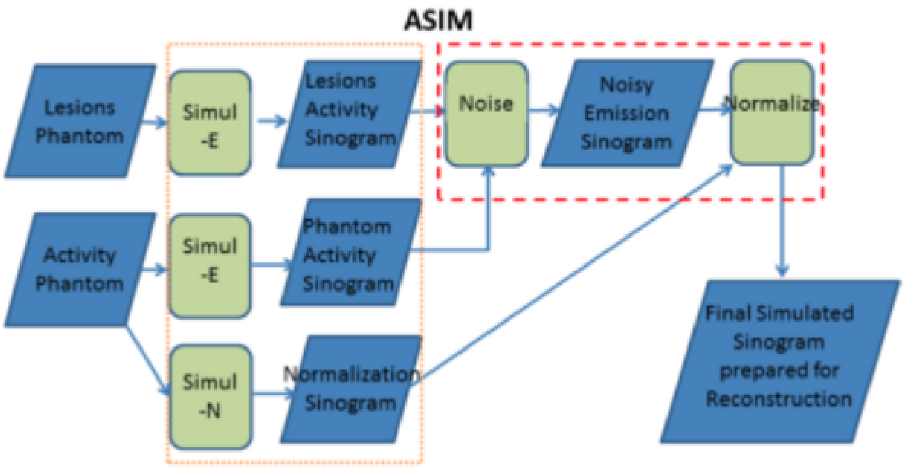

prepare voxelised input file

1. **T1** [180 * 216 * 180 1mm^3]
2. segmenting the original T1 file using volbrain

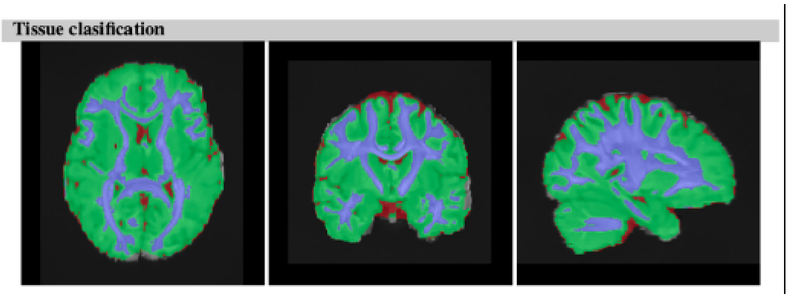

The output file is 1 volume 3D stack with different intensify values correspond to different tissue types: CSF = 1 GM = 2 WM = 3

3. Convert to appropriate format for ASIM voxelised input
   a. assign a uniform value to each tissue segment to represent an expected FDG uptake: grey matter=32767 white matter=8192 CSF=4468

- ASIM input needs to be int16 format, which has a full range of -32767 to 32767
- reference intensity value for FDG uptake: grey matter=44 white matter=11 CSF=6

b. use mrcalc command in MRtrix to perform the intensity descaling, make sure the output data format is uint16be (unsigned int16 big endian)

c. crop to 180 * 180 * 126 1 mm^3

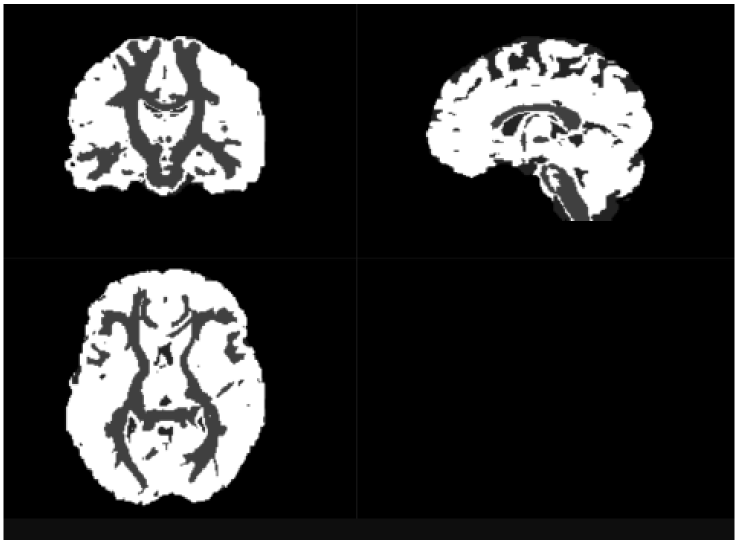

- because the default size is 63 slices for reconstructed PET using STIR

```
#crop x y to be the same dimension --> 180 x 180 x 180
mrconvert t1_ground.nii -coord 1 21:200 -stride 1,2,3 t1_crop.nii

# crop z to 126
mrconvert t1_crop.nii t1_126.nii -coor 2 13:138 -datatype uint16be
```

4. upsample T1 to [0.75 x 0.75 x 1 mm, 240x240x126] and save as .nii file
5. make ASIM input file

a. open .nii file in imageJ

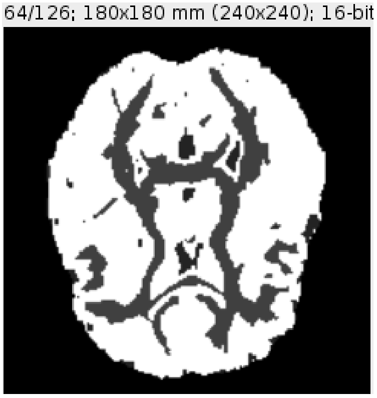

b. image —> transform —> rotate right 180
c. reverse stack order
d. save as raw file with extenstion .yaff

other input templates

modify segmentation

subcortical regions

ASIM simulation

1. prepare .yhdr header file by changing the following parameters only:
   - original_file_name= (the same name as the binary .yaff image file)
   - x_dimension=239 (240-1)
     y_dimension=239 (240-1)
     z_dimension=153

     *STIR recommends using odd number of pixels in all axis
   - scale_factor=0.000153 (1/32767)
   - x_pixel_size_cm=0.075

     y_pixel_size_cm=0.075

     z_pixel_size_cm=0.1
   - image_min=0
     image_max=32767
2. Simulate 3D emission sinogram (inside ASIM_development/Simul/bin folder)

   `./Simul -i t1.yaff -o brain_E.yaff -E -a -m 962 -k -h`

   -E: emission sinogram

   -a: no actuation effect

   -k: arc effect on

   -h: no LORAC

   * by default, emission simulation contains normalisation effects

   * without specifying -d (dimension), the default is 3D sinogram
3. Simulate normalisation file

   `./Simul -i t1.yaff -o brain_nrm.yaff -N -m 962 -k -h`
4. Simulate noise (inside ASIM_development/Noise/bin)

   `./Noise -i brain_E.yaff -t 100000000 -r 20000000 -o brain_noisy.yaff`

   -r (random counts) ~= 20% -t (true counts)

5. Normalisation correction (inside ASIM_development/Normalize/bin)

   ./Normalize -i brain_noisy.yaff -n brain_nrm.yaff -k -o brain_nrmd.yaff

STIR reconstruction

OSMAPOSL

1. prepare STIR header file (only change the following parameters)
   - hdr_E_m962_2D.hs
     - name of data file := brain_nrmd.yaff
2. OSEM.par

```
OSMAPOSLParameters :=
objective function type:= PoissonLogLikelihoodWithLinearModelForMeanAndProjData
PoissonLogLikelihoodWithLinearModelForMeanAndProjData Parameters:=
input file := hdr_E_m962.hs

zero end planes of segment 0:= 0

time frame definition filename :=
time frme number :=
Bin Normalisation type := Chained
Chained Bin Normalisation Parameters:=
END Chained Bin Normalisation Parameters:=

recompute sensitivity := 1
additive sinogram := 0
zoom := 3.0
xy output image size (in pixels) := 240

projector pair type := Matrix
Projector Pair Using Matrix Parameters :=
Matrix type := Ray Tracing
Ray tracing matrix parameters :=
End Ray tracing matrix parameters :=
End Projector Pair Using Matrix Parameters :=
end PoissonLogLikelihoodWithLinearModelForMeanAndProjData Parameters:=

number of subsets:= 12
number of subiterations:= 48

output filename prefix := brain_

save estimates at subiteration intervals:= 48
enforce initial positivity condition:=0
inter-update filter subiteration interval:= 0
inter-update filter type := None
inter-iteration filter subiteration interval:= 4
inter-iteration filter type := None

post-filter type := None

END :=
```

3. visualize reconstructed image in Jupyter Notebook

Read in images

```
FBP=stirextra.to_numpy(stir.FloatVoxelsOnCartesianGrid.read_from_file('brain.hv'))
```

4. STIR built-in quick visualization tool: run command manip_image
5. save .nii in python

```
FBP2=FBP_scaled.astype('float64')
FBP2 = np.flip(FBP2, axis=0)
new_image = nib.Nifti1Image(FBP2, affine=np.eye(4))
nib.save(new_image, '/home/sun/project/FilesForSimulation/ASIM_output/PET_OSEM.nii')
```

6. Re configure strides and assign correct voxel size

```
mrconvert PET_OSEM.nii PET_OSEM2.nii  -axes 2,1,0 -vox 0.75,0.75,1
```

7. open .nii in imageJ and save as Analyze .img .hdr format, this should be in the correct orientation when opened in MRtrix
8. Replace the transform matrix with the transform matrix of the T1 image

```
mrtransform PET_OSEM2-1.img PET_OSEM3.nii -replace /home/sun/project/FilesForSimulation/_final_simulation/t1_126.mif
```

<u>use imageJ to convert .v to mrtrix .nii</u>

-

-
